# Tara and Red Algae Biopolymer as a Film-Forming Substance for Hair Protection

**DOI:** 10.1021/acsomega.5c08778

**Published:** 2026-01-06

**Authors:** Rafaela A. Zito, Rafaela B. Zanin, Leticia Kakuda, Patricia M. B. G. Maia Campos

**Affiliations:** School of Pharmaceutical Sciences of Ribeirão Preto, University of São Paulo, 14040-903 Sào Paulo, Brazil

## Abstract

Considering the impact of environmental aggressors on hair health, the biopolymer derived from tara (*Caesalpinia spinosa*) and red algae (*Kappaphycus alvarezii*) can be applied in the development of hair care formulations due to its film-forming properties. Thus, the objective of this study was to develop hair care formulations containing the biopolymer and evaluate their rheological and texture properties, as well as their film-forming efficacy for hair protection. For this, shampoo, conditioner, and leave-in formulations were developed, and their texture and rheology profile were evaluated during 28 days. Efficacy was analyzed in terms of combability, smoothness, mechanical properties, diameter, and visual aspects before and after the treatment. Formulations with the biopolymer showed a reduced hysteresis area (by 48.17% for the conditioner and 88.09% for the leave-in), which is a rheological parameter directly associated with film formation. In addition, texture parameters decreased, which can improve the sensory properties. The biopolymer interacted with hair keratin via hydrogen bonding, which resulted in a 10.40% reduction in the tensile strength and a 15.85% increase in the fiber diameter. These interactions contributed to film formation on the hair surface, improving shine (by 29.42%) and smoothness (by 21.84%). These results showed that the biopolymer has potential to be applied as a film-forming substance in hair care products by reducing hair–environment interaction.

## Graphical abstract

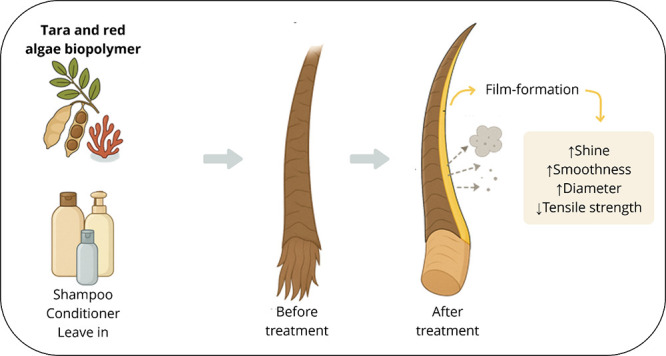

## 1 Introduction

Hair, like skin, plays a vital protective role, acting as a barrier between the body and the external environment.^1,2^ With the modern lifestyle, there has been a greater increase in atmospheric pollution, which intensifies risks to both skin and hair health.^3^ Thus, due to constant exposure to pollutants, skin and hair become highly susceptible to damage caused by these environmental contaminants.^4^

Hair fibers, in particular, are highly vulnerable to air pollution. Their chemical composition favors the adhesion of airborne particles, which can penetrate the keratin structure and trigger oxidative stress and cellular apoptosis in the hair follicle,^5^ and these damages contribute to scalp irritation, such as dandruff, redness, sensitivity, dryness, and pigment degradation, resulting in dull, dry, and lifeless hair.^1,2^

Additionally, hair is exposed to various environmental agents, including humidity, temperature, wind, particulate matter, and solar radiation. UV exposure accelerates the aging of hair fibers and the degradation of melanin. Moreover, the presence of polycyclic aromatic hydrocarbons, combined with sun exposure, intensifies the production of reactive oxygen species within the hair fiber, compromising the integrity of the cell membrane complex and the hair cuticle.^6^

Since the hair fiber does not have active metabolism, it is unable to regenerate after damage caused by external aggressors. This leads to the degradation of the cortex and cuticle, making the fibers porous, stiff, and lacking shine.^1^

Given the multifactorial damage caused by environmental exposure, there is growing interest in developing cosmetic formulations capable of providing protective effects for both skin and hair.^7^ This demand is reinforced by the essential role that healthy hair plays in personal appearance, overall well-being, and quality of life, especially among women.^8,9^

Among the promising strategies to combat pollution-induced damage are antipollution hair products, which aim to form a protective barrier over the tresses, reduce particle adhesion, minimize hair shaft permeability, and seal the cuticle. To achieve this, film-forming agents or antioxidants are commonly used to create a physical shield or neutralize oxidative pollutants.^10^

In recent years, some biopolymers have been studied for hair applications, especially chitosan,^11^ keratin,^12,13^ collagen^14^ and gelatin.^12^ Although these biopolymers have been studied for hair care formulations, respectively, as conditioning agents, repairing agents, rheology modifiers, and styling agents, there is a lack of studies regarding the application of biopolymers in film-forming products specifically designed for daily hair fiber protection. Therefore, the development of formulations that create protective films for everyday hair care remains underexplored. This gap highlights the need for research focused on the film-forming and protective properties of polysaccharides in daily use hair care products that can protect hair fibers from environmental stressors and mechanical damage.

In this context, the biopolymer derived from tara (*Caesalpinia spinosa*) and red algae (*Kappaphycus alvarezii*) shows potential for application in such products, as studies have shown that it forms a protective film and reduces interactions between the skin and environmental pollutants.^7,15,16^ This biopolymer is obtained through the combination of sulfated galactans from *Kappaphycus alvarezii* and galactomannans from *Caesalpinia spinosa*, which produces a polysaccharide-rich biopolymer capable of forming a nonocclusive film-forming network that minimizes interaction with external agents.^15,17^

Therefore, tara and red algae biopolymers have been studied for their film-forming properties and potential application in protective cosmetic formulations for the skin. Previous studies have demonstrated their efficacy in skincare formulations, showing improvements in the skin surface, barrier function, and microrelief.^7,15,16^ Furthermore, substances with film-forming properties, such as the mentioned biopolymer, have also shown potential in protecting hair against oxidative agents, reducing damage caused by bleaching processes.^18^

Despite advances in skin care, studies exploring the use of tara and red algae biopolymers in hair care formulations are scarce, especially regarding fiber protection against external aggressors. Therefore, incorporating tara and red algae biopolymers into hair care formulations could represent a promising strategy for daily protection against environmental aggressors. However, beyond the protective function, consumers also demand products that promote detangling, enhance appearance, and improve shine.^19^ To meet these dual expectations, it is essential to conduct comprehensive physical, mechanical, and biophysical tests on hair tresses to evaluate the properties of the formulations and their effects on hair fibers.

Thus, this study aimed to contribute to the understanding of cosmetic formulations with tara and red algae biopolymers by evaluating their film-forming properties and protective effects on hair. Specifically, we investigated how these biopolymers influence both the rheological and texture profile of the formulations and their film-forming efficacy on hair tresses, providing insights into their potential for developing multifunctional hair care products.

## 2 Materials and Methods

### 2.1 Chemicals

Cocamide DEA, sodium lauryl sulfate, disodium laureth sulfosuccinate, *Kappaphycus alvarezii* extract, and *Caesalpinia spinosa* fruit extract were provided by Galena Quimica e Farmaceutica Ltd. (Campinas, Brazil). Lauryl glucoside, cocamidopropyl betaine, and xylityl sesquicaprylate (and) anhydroxylitol were provided by Chemyunion (Sorocaba, Brazil). Cetrimonium chloride was provided by Chemax Indstria e Comércio (Jandira, Brazil). Cetearyl alcohol, caprylic/capric triglyceride, and behentrimonium methosulfate, (and) cetyl alcohol, (and) butylene glycol were provided by Croda do Brazil Ltd. (São Paulo, Brazil). Glycerin and disodium EDTA were provided by Labsynth Produtos para Laboratórios (São Paulo, Brazil).

### 2.2 Development of the Formulations

Three hair products were developed: shampoo (S), conditioner (C), and leave-in (L). Each formulation had a vehicle control or was supplemented with 1.00% (w/w) tara and red algae biopolymer (SE, CE, and LE), as shown in Table 1.

**Table 1:** Composition of the Shampoo, Conditioner, and Leave-In Formulations (w/w%)^a^

ingredient (INCI^b^name)	SV (%)	SE (%)	CV (%)	CE (%)	LV (%)	LE (%)
cetrimonium chloride			2.00	2.00		
cetearyl alcohol			3.00	3.00		
caprylic/capric triglyceride			1.00	1.00		
behentrimonium methosulfate (and) cetyl alcohol (and) butylene glycol					4.00	4.00
sodium lauryl sulfate	12.00	12.00				
disodium laureth sulfosuccinate	18.00	18.00				
lauryl glucoside	5.00	5.00				
cocamidopropyl betaine	4.00	4.00				
cocamide DEA	2.00	2.00				
glycerin	3.00	3.00	3.00	3.00		
disodium EDTA	0.05	0.05	0.05	0.05	0.05	0.05
xylityl sesquicaprylate (and) anhydroxylitol	1.50	1.50	1.50	1.50	1.50	1.50
distilled water	q.s. 100.00	q.s. 100.00	q.s. 100.00	q.s. 100.00	q.s. 100.00	q.s. 100.00
K. alvarezii extract and C. spinosa fruit extract (biopolymer)		1.00		1.00		1.00

a SV - vehicle shampoo, SE- shampoo added with the biopolymer, CV - vehicle conditioner, CE - conditioner added with the biopolymer, LV - vehicle leave-in, LE - leave-in added with the biopolymer.

b International Nomenclature of Cosmetic Ingredients.

### 2.3 Stability Study

The developed formulations were subjected to preliminary stability tests. Phase separation was analyzed after three centrifugation cycles at 3000 rpm for 30 min (Fanem, São Paulo, Brazil). The organoleptic characteristics of the formulations were visually assessed. Additionally, pH values were determined using a digital pH meter R-TEC-7/2-MP equipped with an electrode for semisolid materials. Samples were stored in glass containers at room temperature (25.00 ± 2.00 °C) and also subjected to thermal stress at 37.00 ± 2.00 °C and 45.00 ± 2.00 °C under controlled humidity conditions (70% relative humidity).^18^ Analyses were conducted in triplicate after 24 h and on days 7, 14, 21, and 28 following formulation preparation.

### 2.4 Rheological Behavior

The conditioner and leave-in formulations were stored at room temperature (25.00 ± 3.00 °C) under controlled humidity and photoperiod conditions to determine their physical stability through an assessment of their rheological behavior. Samples were collected after 24 h and 7, 14, 21, and 28 days of formulation preparation.^20^

A cone–plate DV3T (Brookfield, USA), equipped with spindle CP-52 and RHEOCALCT software, was used to determine the studied parameters. For each analysis, 0.50 ± 0.01 g of the formulation was placed on the plate. Conditioner and leave-in formulations were subjected to a gradual speed increase from 0 to 100 rpm, with 5 s intervals between points. The data generated an ascending and descending curve with 10 points, representing the correlation between “shear stress” and “shear rate”.^18,21^

Rheological curves were integrated using Microcal Origin software, and the hysteresis area was calculated based on the area between the curves. Curves were fitted by using the Ostwald model to determine the flow and consistency indices. Finally, the minimum apparent viscosity was obtained from the highest point of the curve.^21^ For each formulation, all analyses were performed in triplicate.

### 2.5 Texture Profile

The texture profiles of the formulations were determined using a Texture Analyzer – TA.XT Plus (Stable Microsystems, UK) equipped with Exponent 3.0.5.0 software.^20^ Analyses were conducted 24 h after formulation preparation and stored at room temperature.^20,22^

The texture of the formulations was evaluated using the A/BE extruder probe of 40 mm for the conditioner and leave-in formulations and 45 mm for the shampoo formulation. The parameters viscosity index, consistency, firmness, and cohesiveness were assessed.^21^ The container used had a capacity of 125 mL and a diameter of 40 mm, filled to 75% of its capacity. The probe operated under specific conditions, with a return distance of 100 mm and a speed of 20 mm/s.^21,22^ The trigger force used was 2.00 g for shampoo and 14.00 g for conditioner and leave-in. For each formulation, measurements were performed in triplicate.

### 2.6 Hair Tress Standardization Protocol

For the efficacy study, three standardized tresses of virgin brown hair, 20 cm in length and 10 g, were used. Initially, the tresses were cleansed with 3.00% sodium lauryl sulfate solution to obtain baseline measurements. The tresses were numbered 1 to 3: Tress 1 (T1) was used as the untreated control; Tress 2 (T2) was treated with vehicle formulations (SV, CV, and LV); and Tress 3 (T3) was treated with formulations containing the studied biopolymer (SE, CE, and LE).

Tress 1 did not undergo any treatment. Tresses T2 and T3 were treated with 5.00 g of shampoo and 5.00 g of conditioner, applied for 1 min, and rinsed for 1 min. Excess water was removed, and the tresses were partially dried with a hair dryer for 1 min and 40 s. Then, 0.50 g of leave-in was applied for 1 min. Finally, the tresses were fully dried. This treatment cycle was performed three times.

The efficacy test was carried out regarding mechanical properties, fiber diameter, appearance, shine, and combability of the tresses. The measurements were taken at two time points for all tresses. The initial (baseline) measurements (IM) were conducted after the initial cleansing protocol. After the treatment protocol was completed for T2 and T3, the tests were repeated on all tresses, and the final measurements (FM) were conducted. All tests were conducted in a controlled environment with temperatures between 20.00 and 24.00 °C and relative humidity of 40–50%.

### 2.7 Hair Shine

The tresses were evaluated for shine using a Skin Glossymeter GL200 (Courage & Khazaka, Germany) based on the principle of reflectance: more regular surfaces reflect more light and thus appear shinier. The analysis was conducted at temperatures between 20.00 and 24.00 °C, relative humidity of 40–50%, and in a dark room to prevent interference.^23–25^ The measurements were performed five times in each tress.

### 2.8 Tensile Strength and Extensibility

A total of 40 hair fibers with at least 10 cm in length were selected from each hair fiber to assess mechanical properties at two time points: 20 fibers were collected at the initial time (IM) and 20 were collected at the final time, after the treatment with the developed formulations (FM). Fibers were tested using the TA.XT Plus texture analyzer (Stable Microsystems, UK) with a 55 mm gap between supports, a load of 10 N, and a constant speed of 300 mm/min.^23–25^ Stress–strain curves were generated to evaluate the mechanical strength and extensibility of all of the hair samples.

### 2.9 Combability and Smoothness Test

The Texturometer Analyzer TA.XT Plus (Stable Microsystems, United Kingdom) equipment was used to assess hair combability. This instrument has two nonmetallic combs placed on a metal support. When activated, the equipment combs through the hair tress. The software measures the required force for the combs to pass through the hair and calculates their resistance to combing. The study parameter measures the force in gF required to comb each tress. Ten testing cycles were conducted.^23,25^

To assess smoothness, the device has two metal bars, simulating fingers gliding along the hair surface, as on a human scalp. The device measures the force needed for the hair to completely slide between the bars in N. This test was performed five times per tress.^26^

### 2.10 Hair Fiber Diameter and Regularity

The thickness of hair strands was measured using the Visioscan VC 20plus (Courage & Khazaka, Germany). The device provides high-resolution scanning images, and it is possible to measure the thickness of the hair fiber in the picture taken through the equipment’s software.^27,28^ Therefore, 20 hair fibers of each hair fiber were measured in the images provided by the device. The pictures were also visually analyzed to assess the regularity and alignment of the hair tresses based on the images obtained. The measurements were conducted immediately after the washing protocol to avoid interference from the other tests on the regularity of the hair. The diameter of the hair is given in mm.

### 2.11 Statistical Analysis

The experimental data obtained in the studies were analyzed according to a normal distribution using GraphPad Prism 8 (GraphPad Software Inc., USA) and Origin Pro 8 (OriginLab Corp., USA) software. For parametric data, analysis of variance (one-way ANOVA) with Tukey’s post hoc test was used. For nonparametric data, the Kruskal–Wallis test with Dunn’s post-test was used. The confidence level was 95%.

## 3 Results and Discussion

After three centrifugation cycles, the formulations remained homogeneous, with no phase separation observed. In addition, no changes in color, odor, or homogeneity were noted during the study period, even for the formulations subjected to thermal stress (Figure 1). In addition, the shampoo formulations exhibited pH values ranging from 6.50 ± 0.01 to 6.68 ± 0.01, conditioners ranged from 3.67 ± 0.04 to 4.11 ± 0.04, and leave-in formulations ranged from 4.02 ± 0.01 to 4.19 ± 0.01. These pH values obtained are compatible with the expected values for shampoo (between 5.50 and 6.50) and conditioning formulations (between 3.50 and 4.50) as indicated by previous studies.^23^

**Figure 1:**
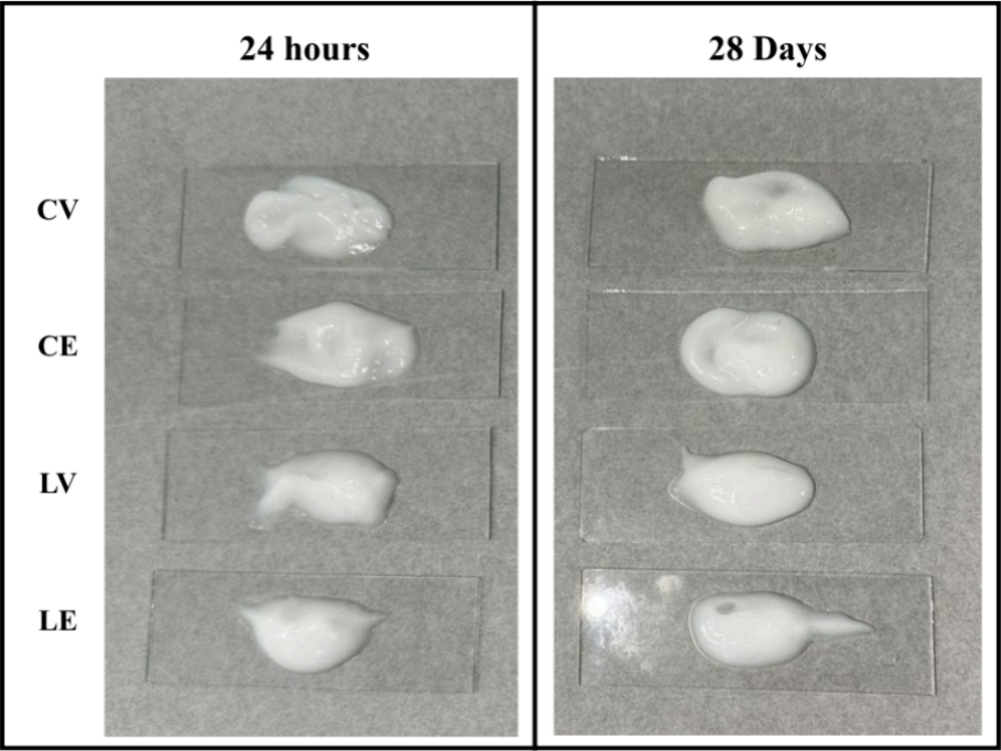
Representative image of the developed emulsions after 24 h and 28 days of the preparation of the formulations.

Regarding rheological behavior, the formulations remained stable during a 28-day study period, with no peaks observed in the rheograms (Figure 2). Also, the developed formulations showed a flow index below 1, ranging from 0.31 to 0.35 for the conditioner and from 0.28 to 0.31 for the leave-in, indicating a non-Newtonian pseudoplastic behavior with thixotropic properties.^7^ These values were maintained for 28 days.

**Figure 2:**
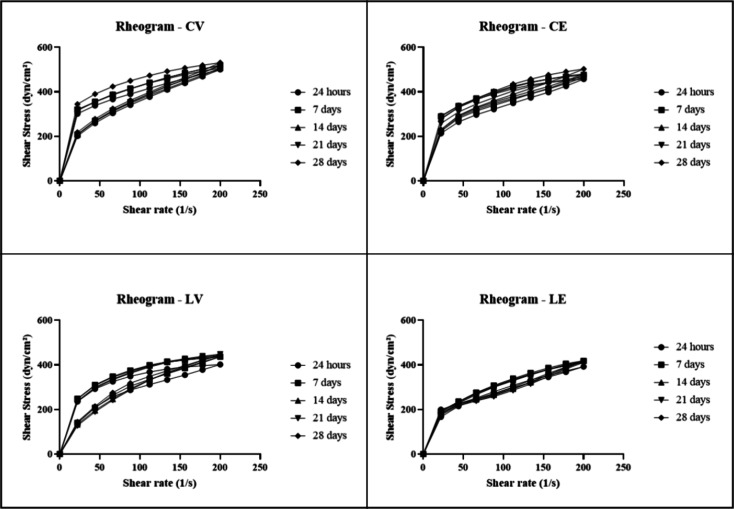
Rheograms of the conditioner and leave-in after 24 h, 7, 14, 21, and 28 days of the preparation of the formulations at room temperature of the vehicle conditioner (CV), conditioner added with the biopolymer (CE), vehicle leave-in (LV), and leave-in added with the biopolymer (LE) (*n* = 3).

Thixotropy is related to the hysteresis area, represented by the space between the ascending and the descending curves of the rheogram. Therefore, lower hysteresis area values indicate a shorter time required for the formulation to recover its viscosity after shear stress caused by application.^20^

The addition of the biopolymer to the conditioner and leave-in formulations led to a significant reduction (*p* < 0.05) in their hysteresis area (Figure 3). Thus, the biopolymer reduces the time needed for the formulations to recover their viscosity after application. This behavior contributes to the type of formulation proposed, as it favors film formation on the applied surface.^7,18^ Thus, the results indicate that the formulations may form a film on the hair surface, creating a protective layer between the hair and potentially harmful environmental agents.

**Figure 3:**
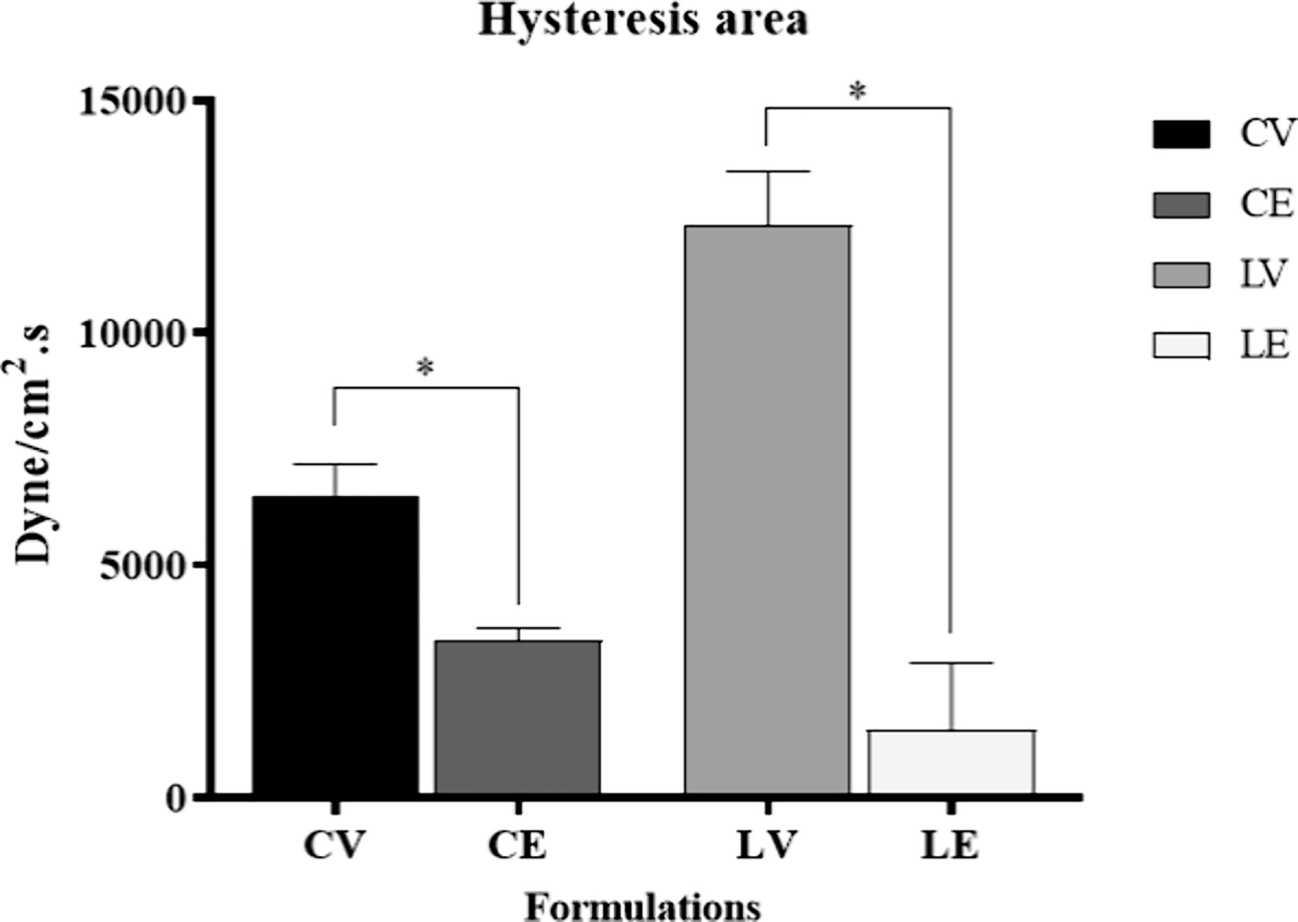
Hysteresis areas of the conditioner and leave-in after 24 h after the preparation at room temperature (*n* = 3). * means the significant difference between the vehicle and formulations added with the active substance (*p* < 0.05).

A non-Newtonian pseudoplastic behavior was observed in formulations containing the biopolymer due to the formation of a polysaccharide network through interactions between hydroxyl groups of galactomannan and sulfated galactans. Under shear, this network breaks down, resulting in the observed behavior.^29^ This happens because there is a reduction in the interaction between polymer chains, leading to a structural breakdown.^30^

Furthermore, thixotropy is essential for the proper performance of conditioner formulations. This behavior is important to ensure that once the product is dispensed from its original packaging, it regains its initial state and behaves like a solid in the palm of the hand. However, when applied to wet hair, the formulation must undergo a reduction in viscosity to facilitate application and rinsing.^31,32^

The same behavior is desired for leave-in formulations, as viscosity recovery plays a crucial role in preventing the product from dripping along the hair after application. The goal of this type of product is to remain deposited on the hair surface, reducing fiber friction.^32^ Therefore, a higher thixotropy is especially important for leave-in formulations, as they are not rinsed off.

Additionally, there was a significant reduction (*p* < 0.05) in the minimum apparent viscosity of CE and LE (Table 2). This indicates that the formulations exhibited greater pseudoplasticity, characterized by a more pronounced decrease in viscosity with increasing shear rate. A higher degree of pseudoplasticity can enhance hair care performance, as it is associated with more uniform lubrication of the hair fiber and the formation of a protective film on its surface.^32^

**Table 2:** Rheological Properties of the Developed Formulations (*n* = 3)^a^

rheological properties	CV	CE	LV	LE
minimum apparent viscosity	224.60 ± 5.80^A^	175.30 ± 2.30^B^	184.90 ± 2.00^A^	126.00 ± 10.30^B^
consistency index	83.64 ± 1.40^A^	52.65 ± 1.70^B^	81.86 ± 0.90^A^	45.02 ± 5.20^B^

a Different letters indicate statistically significant differences between groups (*p* < 0.05).

There was also a significant reduction (*p* < 0.05) in the consistency index of CE and LE compared to their respective vehicles (Table 2). This may increase consumer acceptance of the formulations, as highly consistent products can be more difficult to spread.^18^

Therefore, the rheological parameters suggest that the studied formulations meet the intended goal of exhibiting film-forming characteristics that could contribute to protecting hair against pollutants.

Regarding the texture profile of the formulations, the addition of the biopolymer led to a significant reduction (*p* < 0.05) in the parameters viscosity index, cohesiveness, firmness, and consistency for SE, CE, and LE. High values for these parameters indicate greater difficulty in spreading the formulation;^7^ thus, the addition of the biopolymer to SE, CE, and LE formulations improves their sensory appeal to consumers. These results are consistent with the rheological findings and indicate that the biopolymer altered the formulations’ microstructure.^32^

Regarding the mechanical properties of the hair, the results of this study revealed no significant differences (*p* > 0.05) in the extensibility of the fibers before and after treatment with the evaluated formulations. However, a significant increase (*p* < 0.05) was observed comparing T2 and T3 at FM. Additionally, a significant increase (*p* < 0.05) in fiber diameter was observed for T3 at FM compared to IM and to T2 at FM, while these parameters for T1 and T2 remained unchanged (Figure 4). Finally, a decrease in tensile strength was observed for T3 at FM (*p* < 0.05).

**Figure 4:**
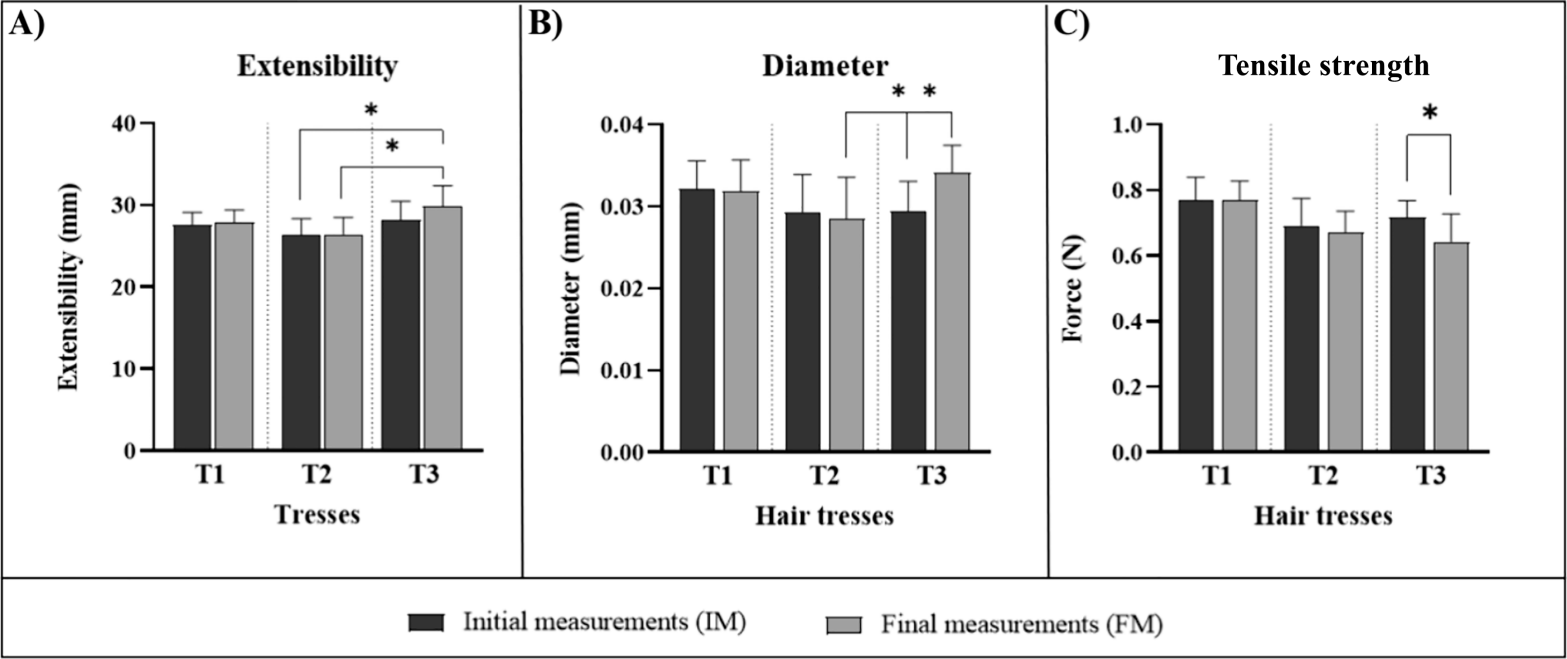
Initial (IM) and final measurements of (A) extensibility, (B) diameter, and (C) tensile strength of tresses after treatment with the studied formulations (*n* = 20). * means the significant difference from baseline (IM) values (*p* < 0.05).

Hair diameter is an important factor in mechanical resistance and is influenced by both the hydration state of hair and its chemical environment. Hair presents hygroscopic properties, being capable of absorbing water.^33^ However, virgin hair exhibits some degree of hydrophobicity due to a lipid layer on its surface, which decreases water absorption, a characteristic that can be altered by cosmetic treatments.^34^

The hydrophobicity of hair is primarily attributed to the presence of fatty acids on its surface, particularly 18-methyleicosanoic acid (18-MEA), a branched-chain fatty acid that plays a crucial role in lubricating hair fibers and reducing friction between them.^35^ In addition, 18-MEA contributes significantly to protecting hair against environmental aggressors.^36^ However, due to its susceptibility to degradation, the loss of 18-MEA can result in increased fragility and a dull appearance of the hair.^35,37,38^ Therefore, applying film-forming agents to the hair surface may help minimize interactions with external aggressors by creating a protective barrier in its surface, preserving the hair structure.

When alterations occur in the hair surface, it becomes more prone to water uptake, resulting in swelling and increased extensibility.^39,40^ Thus, the increase in diameter and extensibility observed for T3 may be attributed to radial swelling of the hair fiber. This is likely due to enhanced water absorption, which is also responsible for the increase in hair extensibility.^33,41^

The mechanical behavior of the hair is determined by its chemical bondings,^42^ especially within its keratin-rich cortex.^24,25,43^ Hydrogen bonds are abundant in the hair, showing great significance in its resistance and extensibility.^40,44^ Hydrogen bonds occur in hair’s α-keratin and can involve side chains of the amino acids that compose this protein or the nitrogen and oxygen atoms of the peptide bonds.^45^ However, in wet conditions, this kind of interaction is annulled due to water interaction with the hair, and it becomes more extensible and less resistant.^40,41,44^

Since hydrogen bonding is relatively weak, it is highly under the influence of external factors, including the cosmetics applied to the hair and humidity.^45^ Therefore, the observed results could be attributed to the chemical bonds between the hair and the biopolymer. Galactomannan, a component of the biopolymer, is rich in hydroxyl groups capable of forming hydrogen bonds with the hair’s keratin structure, as illustrated in Figure 5.^46^ Furthermore, the sulfated galactans also present hydroxyls in their structure, but to a lower degree, which could also perform this kind of bond with the hair.

**Figure 5:**
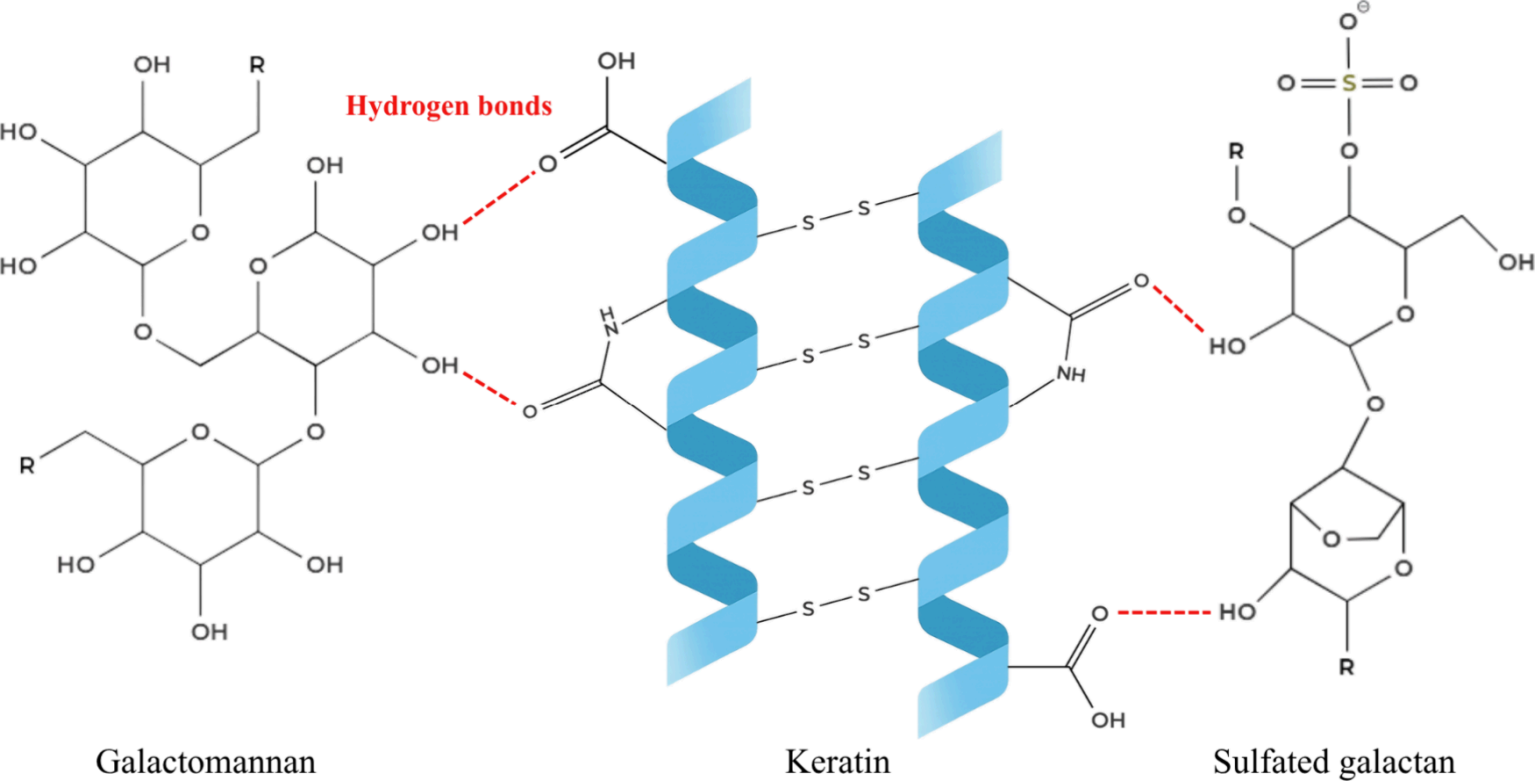
Schematic representation of the interactions between biopolymers and hair keratin. Red dashed lines indicate hydrogen bonds between keratin and galactomannan from tara and sulfated galactan from red algae.

Although these interactions can occur with all the developed products, rinse-off products such as shampoo and conditioner have a limited contact time with the hair.^37^ In contrast, the leave-in formulation, which is applied to dry hair and not rinsed, offers a more prolonged interaction with the fiber.^37^ The longer interaction with the hair could favor the interaction of the biopolymer with the hair, forming new interactions between keratin and the galactomannan and sulfated galactan hydroxyls. Kim et al. described that hydroxyl groups retain moisture, which weakens the hydrogen bonds between keratin molecules, impairing the mechanical strength of the hair.^47^ Therefore, as the biopolymer is rich in hydroxyls, it could promote higher bonding of water with the hair, which modifies the native bonding network within keratin, weakening the original intermolecular associations that contribute to the mechanical strength of the hair.^42^

Overall, the findings suggest that the biopolymer could interact with the hair primarily through hydrogen bonding, altering the original bonding structure that maintains the strength of the fiber. Thus, the observed reduction in tensile strength, increased extensibility, and fiber diameter could be attributed to the break in natural hydrogen bonds in the hair, compromising its rigidity.^41^

However, even if the described interactions can impair hair mechanical properties, they should also promote a higher interaction of the biopolymer with the hair fiber, favoring film formation with the application of the formulations.

An increase in hair shine is expected following treatment, reflecting an improvement in the surface condition of the hair fibers and the alignment of the cuticles due to the expected film formation.^24,25^ This effect was observed in the treatment containing the active substance, where T3 showed a significant increase (*p* < 0.05) in shine at FM, while it also increased compared to T2 at FM. In contrast, T2 presented a significant reduction (*p* < 0.05) in shine (Figure 6).

**Figure 6:**
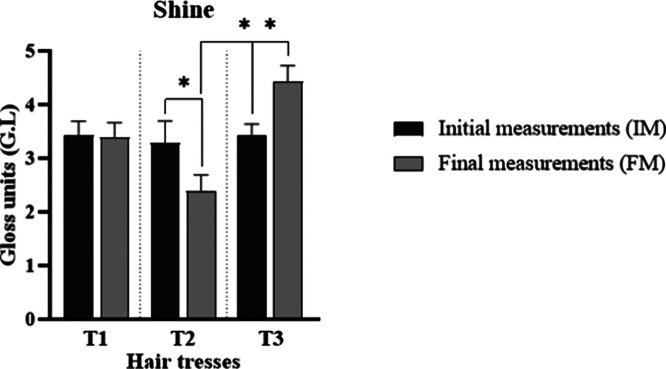
Initial (IM) and final measurements (FM) of shine of tresses after treatment with the studied formulations (*n* = 5). * means the significant difference from initial values (IM) (*p* < 0.05).

Shine is related to the ability of a material to reflect light compared to a perfectly smooth surface. Gloss measurements reflect the topography of the material, with more regular surfaces exhibiting higher gloss values.^48^ These results indicate that treatment with the film-forming substance promoted greater regularity of the hair fibers, whereas the vehicle formulation had the opposite effect. Nagase described that by covering the surface of the hair with polymers, there is a reduction in cuticle inclination, making the hair smoother, which enhances hair shine.^49^

High-resolution images of the hair fibers support the results of the gloss analysis. A remarkable alignment of the hair fibers in tress T3 was observed after the treatment. Conversely, a decrease in the regularity of tress T2 was noted following treatment (Figure 7). It is described in the literature that misaligned hair fibers can lead to a perception of dullness in the hair.^49^ In contrast, aligned fibers exhibit a smooth and shiny appearance.^50^

**Figure 7:**
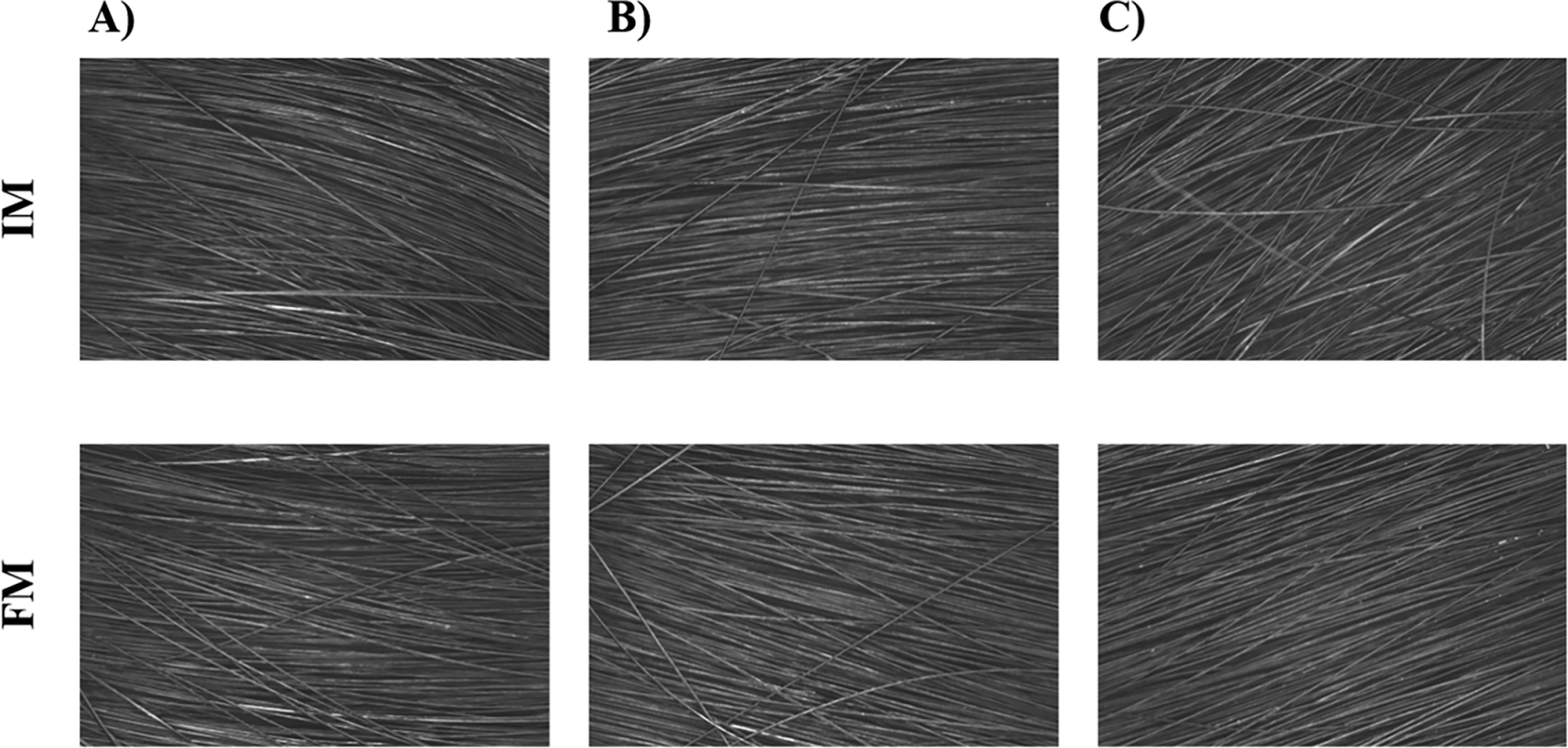
Representative high-resolution images of the hair regularity at initial (IM) and final measurements (FM) time points for the (A) control tress (T1), (B) tress treated with vehicle formulations (T2), and (C) tress treated with the formulation containing the biopolymer. Each image represents a 10 × 8 mm area.

Furthermore, a significant increase (*p* < 0.05) in the smoothness of the T3 tress was observed (Figure 8). This test evaluates the friction on the hair surface and is sensitive to the degree of hair damage. Therefore, the regularity of the hair surface can be assessed, and the formation of a film on the hair may reduce friction between hair fibers, increasing their softness.^19^

**Figure 8:**
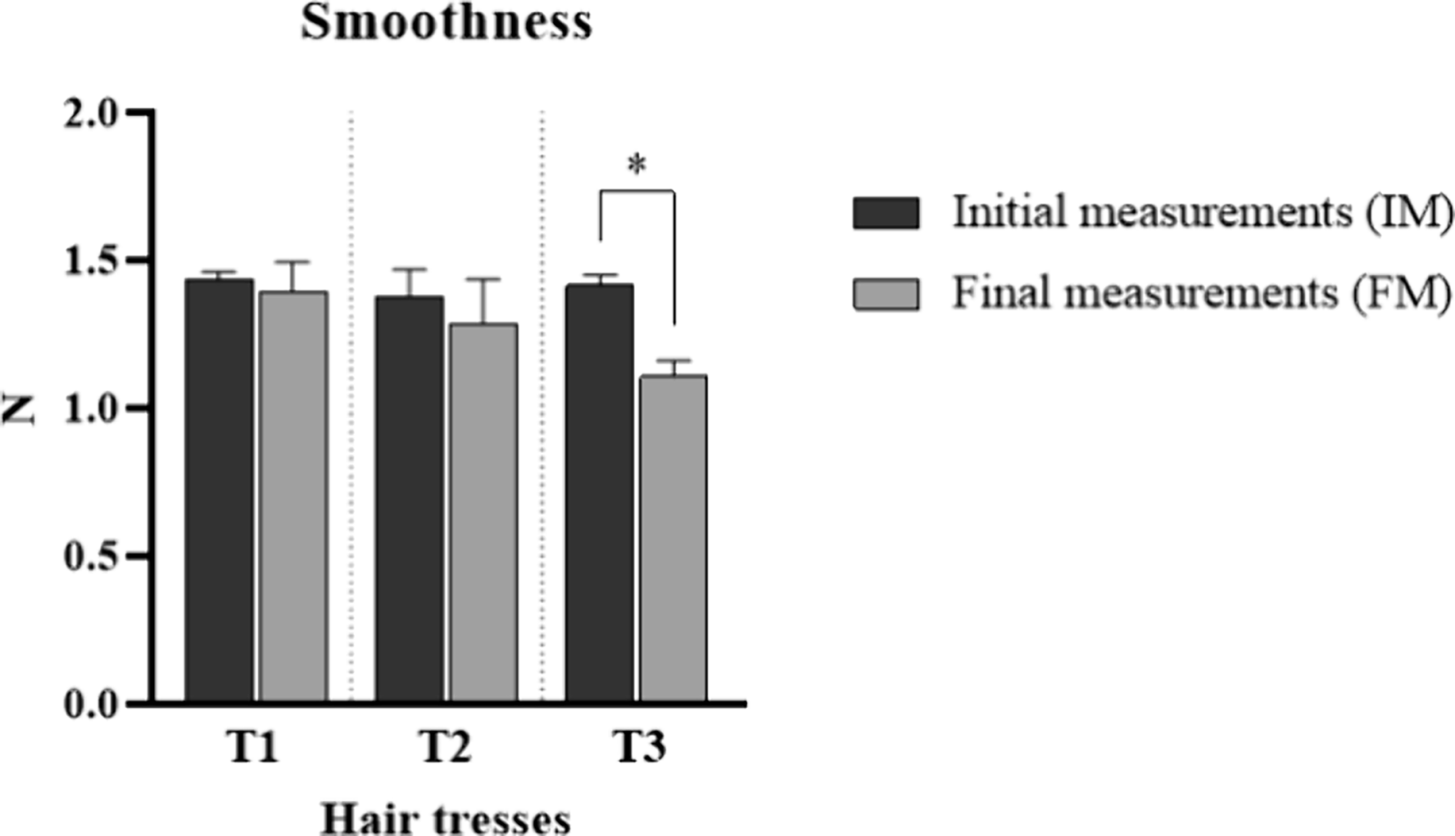
Initial (IM) and final measurements (FM) of smoothness of the tresses after treatment with the developed formulations (*n* = 5). * means the significant difference from baseline (IM) values (*p* < 0.05).

Due to the high molecular weight of polymers, its interaction with the hair fiber can only occur in the hair surface.^37^ Thus, the film formed on the hair surface can lead to the flattening of cuticles over one another, resulting in a smoother fiber,^34,51^ which explains the increased smoothness observed.

Finally, the treatment resulted in a statistically significant decrease (*p* < 0.05) in the force required to comb tresses T2 and T3 (Figure 9). Hair combability is evaluated by the force needed to comb the tresses, with lower values indicating easier combing. The improved combability in both treated tresses can be attributed to the composition of the conditioner and leave-in formulations applied to the tresses.

**Figure 9:**
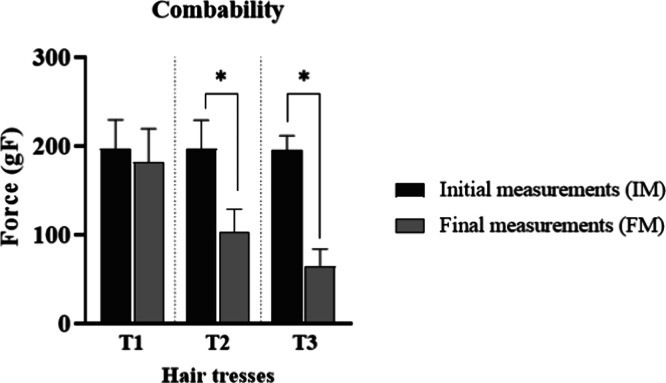
Initial (IM) and final measurements (FM) of combability of the tresses after treatment with the developed formulations (*n* = 10). * means the significant difference from baseline (IM) values (*p* < 0.05).

The reduction in the force required to comb the hair is directly related to the conditioning process. Conditioner and leave-in formulations contain antistatic agents responsible for neutralizing the charges generated on the hair by shampoo application. By minimizing static electricity on the structures, conditioning reduces the force needed for combing. The greater the force required, the higher the risk of mechanical damage to the hair fiber. Thus, a well-conditioned hair requires less force and consequently maintains greater integrity.^24,25,43^

Furthermore, the results for T3 suggest a more pronounced trend in the reduction of this force, highlighting the impact of the biopolymer on this parameter. This effect is attributed to the film-forming characteristic, which provides a protective effect and aligns the cuticles, as previously discussed.^24,25^ Additionally, this effect can be linked to the portion of the biopolymer derived from tara. Tara is composed of galactomannans that are formed of a linear chain of (1–4)-β-D-mannopyranose units with (1–6)-α-D-galactopyranose side units.^29,52^ This structure favors film formation.^52^ Finally, it is also described that galactomannans can act as a conditioning agent on hair,^53^ which can explain the obtained results.

In summary, the efficacy tests suggested an interaction between the biopolymer and α-keratin through hydrogen bonding, which slightly reduced the mechanical strength of the hair fibers. Although a decrease in tensile strength was observed, this can be attributed to the formation of a polymer film on the hair surface that may alter the fiber’s mechanical behavior. Importantly, this film is expected to provide a protective barrier against environmental aggressors, which could otherwise cause more severe structural damage and cumulative weakening of the hair over time.^1,5,6^ Therefore, the protective benefits of the biopolymer coating may offset the initial reduction in tensile strength by preventing further degradation. Moreover, the enhanced deposition of the biopolymer on the hair surface, as evidenced by increased shine and smoothness, provides additional aesthetic benefits that are highly valued in hair care applications.

Finally, these results showed that the biopolymer has potential to be applied in hair care products aimed at the maintenance of natural hair by forming a film in the hair surface, which could reduce hair–environment interaction.

## 4 Conclusions

The addition of the biopolymer in the hair formulations led to a reduction in the hysteresis area of the conditioner and leave-in formulations, which enhanced film formation. Also, it led to a decrease in texture parameters, which can improve the sensory properties. The biopolymer interacted with the hair, resulting in a reduced tensile strength and increased fiber diameter. Although this interaction slightly impaired the mechanical properties, it promoted film formation on the hair surface, leading to improvements in smoothness, shine, and overall appearance. Therefore, this study highlights the film-forming property of the biopolymer on hair, showing its potential to reduce damage caused by environmental aggressors.
